# Effect of Propofol on the Production of Inflammatory Cytokines by Human Polarized Macrophages

**DOI:** 10.1155/2019/1919538

**Published:** 2019-03-17

**Authors:** Tsukasa Kochiyama, Xiaojia Li, Hitoshi Nakayama, Madoka Kage, Yui Yamane, Kenji Takamori, Kazuhisa Iwabuchi, Eiichi Inada

**Affiliations:** ^1^Department of Anesthesiology and Pain Medicine, Juntendo University Graduate School of Medicine, 2-1-1 Hongo, Bunkyo-ku, Tokyo 113-8421, Japan; ^2^Institute for Environmental and Gender-Specific Medicine, Juntendo University Graduate School of Medicine, 2-1-1 Tomioka, Urayasu City, Chiba 279-0021, Japan; ^3^Laboratory of Biochemistry, Juntendo University Faculty of Health Care and Nursing, 2-5-1 Takasu, Urayasu City, Chiba 279-0023, Japan

## Abstract

Macrophages are key immune system cells involved in inflammatory processes. Classically activated (M1) macrophages are characterized by strong antimicrobicidal properties, whereas alternatively activated (M2) macrophages are involved in wound healing. Severe inflammation can induce postoperative complications during the perioperative period. Invasive surgical procedures induce polarization to M1 macrophages and associated complications. As perioperative management, it is an important strategy to regulate polarization and functions of macrophages during inflammatory processes. Although propofol has been found to exhibit anti-inflammatory activities in monocytes and macrophages, it is unclear whether propofol regulates the functions of M1 and M2 macrophages during inflammatory processes. This study therefore investigated the effects of propofol on human macrophage polarization. During M1 polarization, propofol suppressed the production of IL-6 and IL-1*β* but did not affect TNF-*α* production. In contrast, propofol did not affect the gene expression of M2 markers, such as IL-10, TGF-*β*, and CD206, during M2 polarization. Propofol was similar to the GABA_A_ agonist muscimol in inducing nuclear translocation of nuclear factor-E2-related factor 2 (Nrf2) and inhibiting IL-6 and IL-1*β*, but not TNF-*α*, production. Knockdown of Nrf2 using siRNA significantly reduced the effect of propofol on IL-6 and IL-1*β* production. These results suggest that propofol prevents inflammatory responses during polarization of human M1 macrophages by suppressing the expression of IL-6 and IL-1*β* through the GABA_A_ receptor and the Nrf2-mediated signal transduction pathway.

## 1. Introduction

Macrophages are key immune effector cells that are activated by inflammation resulting from tissue damage or infection. During inflammatory processes, monocyte-derived (M0) macrophages undergo polarization to classically (M1) and alternatively (M2) activated macrophages, depending on the local tissue environment [1–3]. M1 macrophages are characterized by the production of high levels of proinflammatory cytokines, an ability to mediate resistance to pathogens, strong microbicidal properties, and promotion of Th1 responses [4]. However, M1 macrophages also contribute to tissue destruction by producing large amounts of reactive nitrogen and oxygen intermediates. For example, invasive surgical procedures in a rat model activated M1 macrophages and increased expression of proinflammatory cytokines, leading to gastric ileus [5]. M1 macrophages recruited during early phases of inflammation promote the production of interleukin- (IL-) 6, IL-1*β*, and tumor necrosis factor- (TNF-) *α*, exacerbating inflammation [1–3]. In contrast, M2 macrophages are characterized by their involvement in immune regulation and homeostatic functions associated with wound healing [6]. Therefore, it would seem to be an important strategy in perioperative management to control macrophage differentiation and immune responses during inflammation and wound healing.

Surgical intervention can induce severe inflammation, leading to postoperative complications such as wound healing disturbance, anastomotic leakage, and infections, with consequent sepsis and multiple organ failure [7]. Suppression of perioperative inflammatory responses can therefore reduce these postoperative complications. Propofol (2,6-diisopropylphenol) is a commonly used intravenous anesthetic agent characterized by the rapid induction of and recovery from anesthesia. Propofol is used for both general anesthesia and sedation in the intensive care unit (ICU). In addition to its anesthetic properties, propofol has been found to suppress the production of IL-6, IL-1*β*, and TNF-*α* by several types of cells [8–12]. Propofol also has hypnotic activity, through the activation of *γ*-aminobutyric acid type A (GABA_A_) receptors [13]. GABA_A_ receptors are pentameric chloride channels usually comprising three different types of subunits [14]. GABA is the major inhibitory neurotransmitter in the central nervous system, and activation of GABA_A_ receptors generally reduces neuronal excitability. Several types of immunological cells, including monocytes, macrophages, and T cells, express GABA_A_ receptors [15–17]. The effects of GABA on T cell functions include suppression of cytokine secretion and modification of cell proliferation [18]. Although GABA_A_ receptor-mediated signaling has been found to affect several immunological functions, the mechanism by which GABA_A_ agonists modulate the functions of those immunological cells remains unclear. Propofol was found to inhibit the chemotaxis and phagocytosis of human monocytes through GABA_A_ receptors [17], as well as to inhibit the production of cytokines by the mouse macrophage cell line RAW 264.7 [19]. However, the effects of propofol on human macrophage polarization and immune responses have not been determined. This study therefore investigated the effects of propofol on macrophage polarization into human M1 and M2 macrophages and on cytokine production by these cells.

## 2. Materials and Methods

### 2.1. Materials

Lymphoprep was obtained from Axis-Shield (Rodelokka, Oslo, Norway). Dulbecco's Modified Eagle's Medium/Nutrient Mixture F-12 (DMEM/F12) was obtained from Invitrogen (Carlsbad, CA, USA). RPMI 1640 medium, LPS from *Escherichia coli* strain O111:B4, and mouse anti-human *β*-actin monoclonal IgG were obtained from Sigma-Aldrich (St. Louis, MO, USA). Recombinant human macrophage colony-stimulating factor (M-CSF), interferon- (IFN-) *γ*, and IL-4 were obtained from R&D Systems (Minneapolis, MN, USA). Propofol was obtained from Wako Pure Chemical Industries (Osaka, Japan). Muscimol (GABA_A_ agonist) was obtained from Abcam (Cambridge, MA, USA). Phycoerythrin- (PE-) conjugated anti-CD86 and anti-CD206 IgGs were obtained from eBioscience (San Diego, CA, USA). Rabbit anti-human nuclear factor-E2-related factor 2 (Nrf2) and mouse anti-human lamin A/C monoclonal IgGs were obtained from Cell Signaling Technology (Danvers, MA, USA). Horseradish peroxidase- (HRP-) conjugated goat anti-rabbit and rabbit anti-mouse IgGs were obtained from Dako (Tokyo, Japan).

### 2.2. Cell Culture and Differentiation

Peripheral blood was obtained from healthy volunteers, all of whom provided written informed consent. The study protocol was approved by the Local Ethics Committee of Juntendo University Urayasu Hospital, and the study was registered with the University Hospital Medical Information Network (UMIN) (registration number UMIN000019625). Peripheral blood mononuclear cells (PBMCs) were separated from blood samples using Lymphoprep according to the manufacturer's protocol. The PBMCs were suspended in DMEM/F12, plated onto 12-well tissue culture plates at a density of 4.0 × 10^6^/ml, and cultured for 3 hr at 37°C. Adherent cells (monocytes) were differentiated into macrophages (defined as M0 macrophages) by incubation in RPMI 1640 supplemented with 20% fetal bovine serum (FBS) and 100 ng/ml M-CSF in 12-well plates for 7 days. M1- and M2-polarized macrophages were obtained by culturing M0 macrophages for 18 hr in RPMI 1640 supplemented with 5% FBS in the presence of 100 ng/ml LPS plus 20 ng/ml IFN-*γ* or 20 ng/ml IL-4, respectively [20]. M1 macrophages were characterized by expression of CD86 and the production of proinflammatory cytokines, such as IL-6, IL-1*β*, and TNF-*α* [21]. In contrast, M2 macrophages, generated by polarization with IL-4, were characterized by the expression of CD206 and production of anti-inflammatory cytokines, such as IL-10 and transforming growth factor- (TGF-) *β*1 [22]. Under these experimental conditions, M1-polarized macrophages expressed higher levels of CD86, IL-6, IL-1*β*, and TNF-*α* mRNAs than did M0 and M2-polarized macrophages, as shown by qRT-PCR assays (Supplementary Figure 1). In contrast, M2-polarized macrophages expressed higher levels of CD206 mRNA than did M0 and M1-polarized macrophages. Flow cytometric analysis showed that surface expression of CD86 was higher on M1-polarized macrophages than on M0 macrophages and that surface expression of CD206 was higher on M2-polarized macrophages than on M0 macrophages (Supplementary Figure 1).

Human monocytic leukemia THP-1 cells (ATCC, Manassas, VA, USA) resemble primary monocytes and macrophages in morphology and differentiation property. THP-1 cells exposed to phorbol-12-myristate-13-acetate (PMA) start to adhere to culture plates and begin to differentiate into a macrophage-like phenotype; these cells are generally used to study human macrophage functions [23]. THP-1 cells were differentiated into macrophage-like (M0 THP-1) cells by incubation for 3 days with 200 nM PMA in RPMI 1640 supplemented with 5% FBS, penicillin (100 IU/ml), and streptomycin (100 *μ*g/ml) [23]. M0 THP-1 cells were polarized into M1 or M2 macrophage-like (M1 THP-1 or M2 THP-1) cells by incubation with 100 ng/ml LPS plus 20 ng/ml IFN-*γ* or 20 ng/ml IL-4, respectively [3]. M1 THP-1 cells expressed higher levels of IL-6, TNF-*α*, and CD86 mRNAs than did M0 and M2-polarized THP-1 cells, whereas M2 THP-1 cells expressed higher levels of CD206 mRNA than did M0 and M1-polarized THP-1 cells (Supplementary Figure 2). M0 macrophage-like THP-1 cells were confirmed as being appropriately polarized to M1 or M2 macrophage-like cells under these experimental conditions.

In general, clinical blood concentration of propofol used for general anesthesia ranges from 2.0–4.0 *μ*g/ml (11.2–22.4 *μ*M). Mean *in vivo* plasma concentrations of propofol required for moderate sedation (slow response to painful stimulation) and deep sedation (no response to painful stimulation) are 0.5 ± 0.2 *μ*g/ml (2.8 ± 1.1 *μ*M) and 1.4 ± 0.6 *μ*g/ml (7.8 ± 3.3 *μ*M), respectively [24]. We examined the effects of propofol in these clinical concentration ranges on M1 polarization. To evaluate the effects of propofol on differentiation and inflammatory responses during macrophage polarization, M0 macrophages were polarized to M1 or M2 macrophages in the presence of propofol (1–5 *μ*M) or solvent alone (0.05% DMSO). In some experiments, M0 macrophages were polarized to M1 macrophages in the presence of muscimol (100 *μ*M). M0 THP-1 cells were polarized into M1 or M2 THP-1 cells in the presence of propofol (25–100 *μ*M), muscimol (100 *μ*M), or solvent alone. Under these experimental conditions, propofol and muscimol had little effect on the viability of polarized macrophages and THP-1 cells (>95% by trypan blue staining).

### 2.3. Quantitative Real-Time RT-PCR (qRT-PCR) Assays

qRT-PCR assays were performed as described previously [21]. In brief, total RNA was extracted and purified from cells using RNeasy Mini Kits (Qiagen, Valencia, CA, USA), and cDNA was synthesized from total RNA preparations using an ExScript RT-PCR kit (Takara Bio, Shiga, Japan). cDNA was amplified using an ABI 7900HT Sequence Detection System (Applied Biosystems, Foster City, CA, USA) and specific primers (Takara, Table 1). To determine relative cDNA concentrations, standard curves were plotted with sequential 10-fold dilutions of cDNA synthesized from 500 ng QPCR Human Reference Total RNA (Stratagene, La Jolla, CA, USA). The level of expression of each gene was normalized relative to that of *β*-actin (internal control).

### 2.4. Enzyme-Linked Immunosorbent Assays (ELISA)

Concentrations of IL-6, IL-1*β*, and TNF-*α* in culture supernatants from polarized macrophages were determined by ELISA using ELISA MAX kits (BioLegend, San Diego, CA, USA), according to the manufacturer's instructions. Concentrations of IL-10 and TGF-*β*1 in culture supernatants from polarized macrophages were determined by ELISA using DuoSet ELISA kits (R&D Systems, Minneapolis, MN, USA), according to the manufacturer's instructions.

### 2.5. Flow Cytometric Analysis

M0, M1, and M2 macrophages pretreated with an FcR blocker (Miltenyi Biotec, Bergisch Gladbach, Germany) were immunostained with PE-conjugated anti-CD86, anti-CD206, or isotype control antibodies for 30 min at 4°C. Antigen expression levels on cell surfaces were measured by flow cytometry (FACSCalibur, BD Biosciences, Franklin Lakes, NJ, USA).

### 2.6. Nuclear Extraction and Western Blotting Analysis

Nuclear proteins were extracted from THP-1 cells using a Nuclear Extract Kit (Active Motif Japan, Tokyo). Aliquots containing 15 *μ*g protein were separated by 7.5% SDS-PAGE and transferred to PVDF membranes (Millipore Corp., Bedford, MA, USA). The membranes were incubated with an anti-Nrf2 antibody (concentration, 1 : 2000) overnight at 4°C, washed with TBS-T (10 mM Tris-HCl (pH 8.0) and 150 mM NaCl containing 0.05% Tween 20), and incubated with an HRP-conjugated secondary antibody (concentration, 1 : 4000). The membranes were subsequently stripped by incubation with stripping buffer (62.5 mM Tris-HCl (pH 6.8), 100 mM *β*-mercaptoethanol, and 2% SDS) for 30 min at 55°C and incubated with an anti-lamin A/C antibody (concentration, 1 : 2000). Bands detected with a SuperSignal reagent (Thermo Fisher/Pierce) were scanned, and chemiluminescence signal intensities were quantified using ImageJ software (U.S. National Institutes of Health, http://rsb.info.nih.gov/ij/).

### 2.7. Transfection of Short Interfering RNA (siRNA)

Short interfering RNAs (siRNAs) were obtained from Dharmacon (Lafayette, CO, USA). THP-1 cells were transfected with human Nrf2 siRNA (L-003755-00-0005) or nontargeting control siRNA (D-001810-10-05), each at a final concentration of 100 nM, using Nucleofector Kit V (Amaxa Biosystems, Cologne, Germany), according to the manufacturer's protocol with slight modifications. Following transfection, the cells were incubated with PMA for 72 hr, collected, lysed in 100 *μ*l lysis buffer (10 mM Tris-HCl (pH 7.4), 50 mM NaCl, 10 mM NaF, 2 mM Na_3_VO_4_, 1 mM PMSF, and 1% Triton X-100 with 1/20 *v*/*v* Complete), and sonicated for 10 s with an ultrasonic disruptor (Sonifier model 250, Branson Ultrasonics, Danbury, CT, USA). The resulting lysates were subjected to immunoblotting analysis. Nrf2 expression was measured by western blotting as above, with *β*-actin as the loading control. Detected bands were scanned, and intensities of chemiluminescence signals were quantified by ImageJ software. PMA-differentiated siRNA-transfected cells were further polarized into M1 macrophages in the presence or absence of propofol (50 *μ*M), and IL-6, IL-1*β*, and TNF-*α* concentrations in supernatants were measured by ELISA.

### 2.8. Statistical Analysis

Values are expressed as the mean ± SD. Differences between two groups were analyzed by Wilcoxon-Mann-Whitney test, whereas differences among multiple groups were analyzed by one-way analysis of variance (ANOVA), followed by Bonferroni's post hoc test. All statistical analyses were performed using the GraphPad Prism software program V. 6.00 (GraphPad Software, La Jolla, CA, USA), with *P* < 0.05 defined as statistically significant.

## 3. Results

### 3.1. Propofol Suppresses IL-6 and IL-1*β* Expression in M1 Macrophages but Does Not Affect M2 Polarization

**Figure 1:**
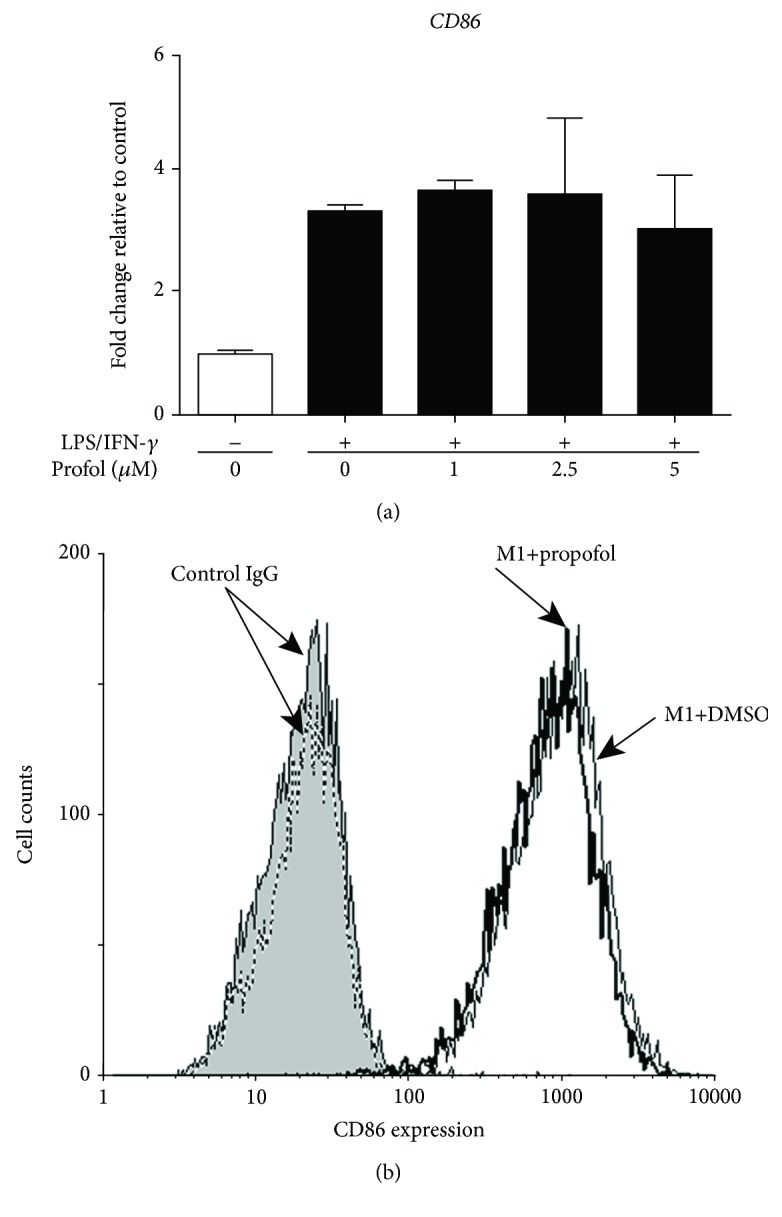
Propofol had no effect on CD86 mRNA and cell surface expression in M1 macrophages. M0 macrophages were polarized to M1 macrophages in the presence of 0.05% DMSO (solvent control) or propofol (1–5 *μ*M). (a) qRT-PCR assays of CD86 mRNA levels. Data were normalized relative to *β*-actin mRNA (internal control) and presented as mean ± SD (*n* = 3 per group). (b) Flow cytometric analysis of CD86 surface expression on M1 macrophages treated with propofol (thick line) or DMSO (thin line) and on M1 macrophages incubated with isotype-matched control IgG and propofol (gray-filled line) or DMSO (dashed line).

**Figure 2:**
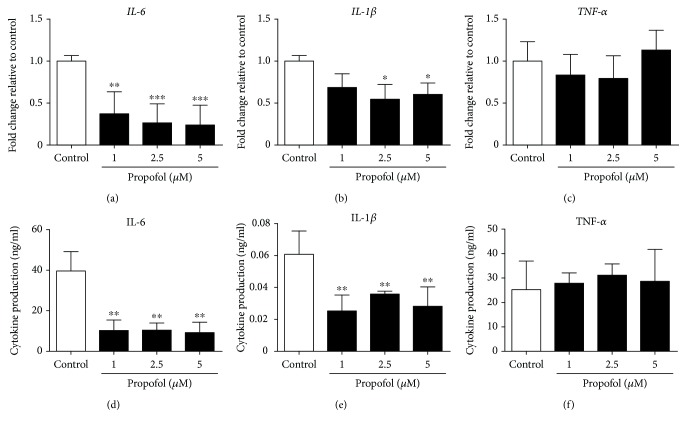
Propofol reduced IL-6 and IL-1*β* gene expression and protein production in M1 macrophages. M0 macrophages were polarized to M1 macrophages in the presence of 0.05% DMSO (solvent control) or propofol (1–5 *μ*M). (a–c) qRT-PCR assays of IL-6, IL-1*β*, and TNF-*α* mRNA levels in M1 macrophages. Data were normalized relative to *β*-actin mRNA (internal control) and presented as mean ± SD (*n* = 3 per group). (d–f) ELISA measurements of IL-6 (d), IL-1*β* (e), and TNF-*α* (f) secreted by M1 macrophages. Data are presented as mean ± SD (*n* = 3 per group). ^∗^ *P* < 0.05, ^∗∗^ *P* < 0.01, and ^∗∗∗^ *P* < 0.001 compared with control cells by one-way ANOVA with Bonferroni's post hoc test.

First, we evaluated the effect of propofol on cytokine production during M1 polarization of macrophages and M0 THP-1 cells. Under our experimental conditions, propofol had no effect on CD86 mRNA levels or cell surface expression of CD86 on macrophages during M1 polarization (Figures 1(a) and 1(b)). Under these conditions, propofol significantly reduced the expression of IL-6 and IL-1*β* mRNAs by M1 macrophages but did not affect the expression of TNF-*α* mRNA by these cells (Figures 2(a)–2(c)). Propofol had similar effects on M0 THP-1 cells during M1 polarization, significantly reducing IL-6 and IL-1*β* mRNA expression, but having no effect on TNF-*α* mRNA expression, by M1 THP-1 cells (Supplementary Figure 3A-C).

The effects of propofol on the production of proinflammatory cytokines by M1 macrophages were examined by ELISA. During M1 polarization, propofol significantly inhibited the release of IL-6 and IL-1*β*, but not of TNF-*α*, from macrophages (Figures 2(d)–2(f)). Similar results were observed with M1 THP-1 cells. Propofol significantly inhibited the secretion of IL-6 and IL-1*β*, but not of TNF-*α*, from M1 THP-1 cells (Supplementary Figure 3D-F).

**Figure 3:**
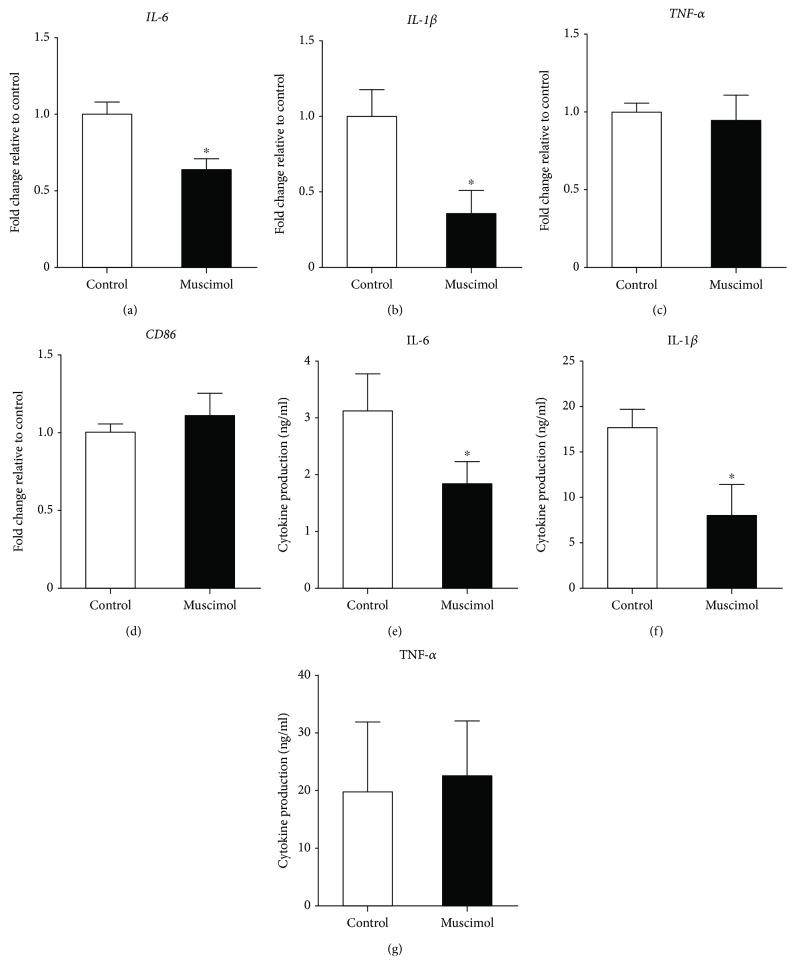
Muscimol reduced IL-6 and IL-1*β* gene expression and protein production in M1 macrophages. M0 macrophages were polarized to M1 macrophages in the absence or presence of muscimol (100 *μ*M). (a–d) qRT-PCR assays of IL-6, IL-1*β*, TNF-*α*, and CD86 mRNA. Data were normalized relative to *β*-actin mRNA (internal control) and presented as mean ± SD (*n* = 4 per group). (e–g) ELISA measurements of IL-6 (e), IL-1*β* (f), and TNF-*α* (g) secreted by M1 macrophages. Data are presented as mean ± SD (*n* = 4 per group). ^∗^ *P* < 0.05 compared with control cells by Wilcoxon-Mann-Whitney test.

Propofol has been shown to directly bind to GABA_A_ receptors [13], which are present on human monocytes and THP-1 cells [17]. In mouse peritoneal macrophages, the GABA_A_ agonist muscimol was found to inhibit IL-1*β* production [25], and GABA was observed to suppress the gene expression of IL-6 and IL-12 by LPS-stimulated cells [16]. We therefore compared the effects of muscimol and propofol on the M1 polarization of M0 macrophages. Similar to propofol, muscimol significantly reduced the expression of IL-6 and IL-1*β* mRNAs but had no effect on the expression of TNF-*α* and CD86 mRNAs (Figures 3(a)–3(d)). Muscimol also significantly inhibited the release of IL-6 and IL-1*β*, but not of TNF-*α*, from macrophages (Figures 3(e)–3(g)).

**Figure 4:**
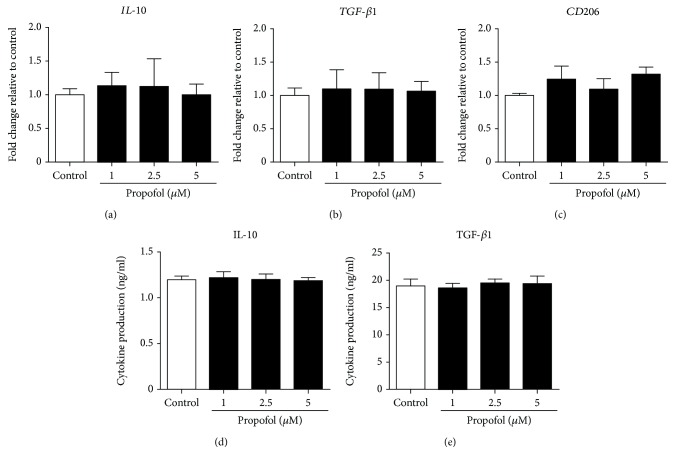
Propofol had no effect on IL-10, TGF-*β*1, and CD206 gene expression and protein production in M2 macrophages. M0 macrophages were polarized to M2 macrophages in the presence of 0.05% DMSO (solvent control) or propofol (1–5 *μ*M). (a–c) qRT-PCR assays of IL-10, TGF-*β*1, and CD206 mRNA. Data were normalized relative to *β*-actin mRNA (internal control) and presented as mean ± SD (*n* = 3 per group). ELISA measurements of IL-10 (d) and TGF-*β*1 (e), secreted by M2 macrophages. Data are presented as mean ± SD (*n* = 3 per group). Statistical comparisons were analyzed using one-way ANOVA with Bonferroni's post hoc test.

Next, we examined the effect of propofol on M2 polarization of macrophages and M0 THP-1 cells. Under our experimental conditions, propofol had no effect on the expression of IL-10, TGF-*β*1, and CD206 mRNAs during M2 polarization of M0 macrophages (Figures 4(a)–4(c)) or by M2 THP-1 cells (Supplementary Figure 4A-C). IL-10 and TGF-*β*1 production from M2 macrophages (Figures 4(d) and 4(e)) and M2 THP-1 cells (Supplementary Figure 4D, E) was not affected by propofol administration.

### 3.2. Propofol and Muscimol Induce Nuclear Translocation of Nrf2 during M1 Polarization

**Figure 5:**
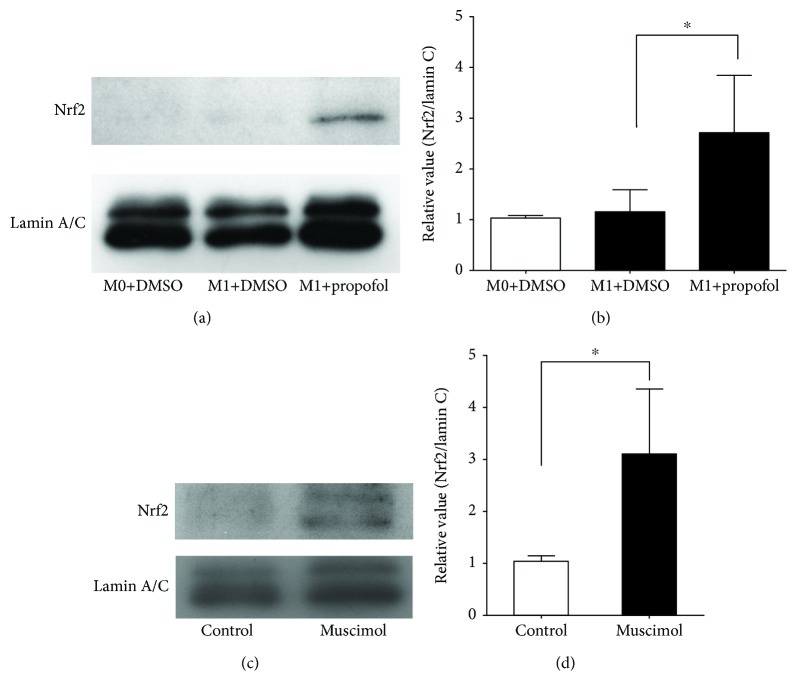
Propofol and muscimol enhanced nuclear translocation of Nrf2 in M1 THP-1 cells. (a) Immunoblotting analysis of the effects of propofol (50 *μ*M) on nuclear translocation of Nrf2. (b) Densitometric analysis of bands in (a). (c) Immunoblotting analysis of the effects of muscimol (100 *μ*M) on nuclear translocation of Nrf2. (d) Densitometric analysis of bands in (c). Data were normalized relative to lamin C (internal control for nuclear proteins) and presented as mean ± SD of four independent experiments. ^∗^ *P* < 0.05 compared with control cells by one-way ANOVA with Bonferroni's post hoc test or by the Wilcoxon-Mann-Whitney test.

Nrf2 is a transcription factor that mediates various physiological responses [26]. In a rat liver transplant model, propofol was associated with amelioration of oxidative stress-induced acute lung injury via strong activation of Nrf2 [27]. In rat cardiac H9c2 cells, propofol induced the nuclear translocation of Nrf2 and exerted antioxidative effects [28]. While Nrf2 is known for its antioxidant activity, Nrf2 activation was found to suppress the production of IL-6 and IL-1*β*, but not of TNF-*α*, by mouse M1 macrophages [29]. Therefore, we examined the effects of propofol and muscimol on the nuclear translocation of Nrf2 in M1 THP-1 cells. We found that both propofol (Figures 5(a) and 5(b)) and muscimol (Figures 5(c) and 5(d)) significantly increased the nuclear translocation of Nrf2 in M1 THP-1 cells.

### 3.3. Nrf2 Mediates the Inhibitory Effect of Propofol on IL-6 and IL-1*β* Production during M1 Polarization

**Figure 6:**
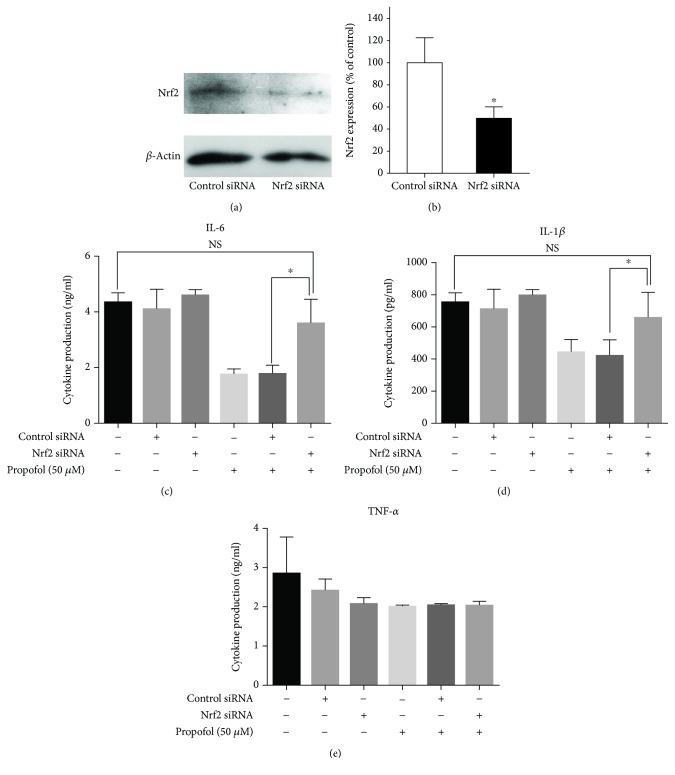
siRNA knockdown of Nrf2 significantly reduced the anti-inflammatory effects of propofol in M1 THP-1 cells. (a) Immunoblotting analysis of Nrf2 expression. THP-1 cells were transfected with control nontarget or Nrf2 siRNA and treated with PMA for 72 hr. (b) Densitometric analysis of bands in (a). Data were normalized relative to *β*-actin (internal control) and presented as mean ± SD of four independent experiments. ^∗^ *P* < 0.05 compared with control cells by the Wilcoxon-Mann-Whitney test. (c–e) Effects of Nrf2 siRNA on IL-6, IL-1*β*, and TNF-*α* production by M1 THP-1 cells in the absence or presence of propofol. PMA-differentiated siRNA-transfected cells were further polarized into M1 macrophages in the presence of 0.05% DMSO (solvent control) or propofol (50 *μ*M). IL-6, IL-1*β*, and TNF-*α* concentrations in supernatants were measured by ELISA. ^∗^ *P* < 0.05 compared with cells transfected with nontarget siRNA by one-way ANOVA with Bonferroni's post hoc test. NS = not significant.

In resting cells, Nrf2 is constitutively degraded in a Kelch-like ECH-associated protein 1- (Keap1-) dependent manner [26]. Oxidative or electrophilic stress leads to the dissociation of Nrf2-Keap1 complexes, the accumulation of Nrf2, and the translocation of Nrf2 into the nucleus [30]. To determine whether propofol suppresses IL-6 and IL-1*β* production by activating Nrf2, Nrf2 expression was knocked down in M1 THP-1 cells by Nrf2-specific siRNA. THP-1 cells were transfected with control or Nrf2 siRNA and treated with PMA for 72 hr. Compared with control siRNA, transfection with Nrf2 siRNA reduced Nrf2 expression by 49.1% (Figure 6(b)). During M1 polarization of transfected cells in the presence or absence of 50 *μ*M propofol, Nrf2 siRNA significantly blocked the ability of propofol to inhibit IL-6 and IL-1*β* production, with control levels observed (Figures 6(c) and 6(d)). However, regardless of propofol treatment, Nrf2 siRNA did not affect TNF-*α* production compared with control levels (Figure 6(e)).

To evaluate the effects of propofol and muscimol on Nrf2-mediated antioxidant activity, we assayed the expression of antioxidant genes regulated by Nrf2, such as NAD(P)H quinone dehydrogenase 1 (NQO1), glutamate-cysteine ligase catalytic subunit (GCLC), and heme oxygenase 1 (HMOX1) mRNAs in M0 macrophages during M1 polarization. Neither propofol (Supplementary Figure 5A-C) nor muscimol (Supplementary Figure 5D-F) had any effect on the expression of NQO1, GCLC, and HMOX1 mRNAs during M1 polarization.

## 4. Discussion

The results of the present study demonstrated that propofol significantly inhibited the production of IL-6 and IL-1*β*, but not of TNF-*α*, by human M1 macrophages. Propofol induced nuclear translocation of Nrf2, suppressing the expression of IL-6 and IL-1*β*. In contrast to its effects on M1 macrophages, propofol did not affect the expression of genes encoding anti-inflammatory cytokines during M2 polarization. M2 macrophages are involved in anti-inflammatory and homeostatic functions associated with wound healing, fibrosis, and tissue repair [1]. Our finding that propofol did not affect the function of M2 macrophages suggests that propofol likely suppresses M1 macrophage-induced inflammatory responses without altering M2 macrophage functions such as anti-inflammatory effects and tissue repair.

In the case of THP-1 cells, higher concentration of propofol was needed to suppress the gene expression and production of IL-6 and IL-1*β* (Supplementary Figure 3D-F). THP-1 is a type of leukemia cell line that can be differentiated into macrophage-like cells by treatment with PMA. The malignant background of THP-1 cells might possibly result in different sensitivities and responses compared to those of primary monocytes [31]. It was demonstrated that IC_50_ values of three different kinds of TNF-*α* secretion inhibitors on TNF-*α* production from human monocytes differed from those of THP-1 cells [32]. Therefore, higher concentrations of propofol may be needed to suppress the production of proinflammatory cytokines by M1 THP-1 cells than by primary M1 macrophages.

Hydrophobic molecules such as steroid hormones and thyroid hormones can diffuse directly across cell plasma membranes and bind to intracellular receptors and then directly regulate expression of receptor genes [33]. Since propofol is a hydrophobic molecule, it could conceivably enter macrophages and affect the activities of transcription factors and signal transduction molecules responsible for the production of IL-6 and IL-1*β*. However, propofol has been demonstrated to be able to directly bind to GABA_A_ receptors [13]. Although the GABA_A_ receptor agonist muscimol is a hydrophilic compound, which cannot enter the cytoplasm of a cell, muscimol inhibited IL-6 and IL-1*β* expression and induced Nrf2 translocation into the nucleus as well as propofol, suggesting that propofol might regulate IL-6 and IL-1*β* through GABA_A_ receptors.

In the present study, propofol and muscimol induced the nuclear translocation of Nrf2 during M1 polarization of human macrophages and inhibited the production of IL-6 and IL-1*β*, but not of TNF-*α*. Induction of Nrf2 leads to its accumulation in the cytoplasm, followed by its translocation into the nucleus [30]. Inflammatory responses, including cytokine production, were induced in THP-1 cells by *Mycoplasma pneumoniae*-derived lipid-associated membrane proteins (LAMPs) [33]. These responses were significantly elevated in LAMP-stimulated Nrf2-silenced THP-1 cells, indicating that Nrf2 negatively regulates inflammatory responses of macrophages. Knockdown with Nrf2-specific siRNA significantly reduced the inhibitory effects of propofol on IL-6 and IL-1*β* production by M1 macrophages (Figure 6). In resting cells, Nrf2 is constitutively degraded in a Keap1-dependent manner [26]. Keap1 is an adaptor protein for a Cul3-based ubiquitin E3 ligase [34]. Keap1 binds to Nrf2 and promotes the ubiquitination of this protein for degradation by proteasomes. The knockout of the Keap1 gene resulted in accumulation of Nrf2 in the cytoplasm of cells [29]. Macrophage production of IL-6 and IL-1*β*, but not of TNF-*α*, was suppressed during M1 polarization of conditional Keap1 gene-knockout mice through binding to proximal regulatory regions without oxidative stress [29]. Therefore, it is likely that Nrf2 is involved in transcriptional regulation for IL-6 and IL-1*β* gene expression, while TNF-*α* gene expression is not regulated by Nrf2 during M1 macrophage polarization. Taken together, these findings indicate that propofol induces cytoplasmic accumulation and nuclear translocation of Nrf2 through activation of GABA_A_ receptors, resulting in the inhibition of IL-6 and IL-1*β* expression during M1 macrophage polarization.

There are several studies investigating the effects of propofol on TNF-*α* modulation in several types of cells [12, 35, 36]. LPS-induced inflammatory reactions, including TNF-*α* production, have been demonstrated to be suppressed by propofol using the mouse cell line RAW 264.7 [12, 35] and canine PBMCs [36]. On the other hand, we demonstrated that propofol suppressed human M1 macrophage-induced genes, such as IL-6 and IL-1*β*, but not TNF-*α*. We induced M1 macrophage polarization using the combination of LPS and IFN-*γ*. As also described in the present study, gene expression of TNF-*α* is not regulated by Nrf2 during M1 polarization [29]. It seems, therefore, that the signaling pathways involved in TNF-*α* production during M1 polarization of human macrophages are different from those in TNF-*α* production by LPS-stimulated macrophage-like cells and PBMCs.

Although Nrf2 has been shown to activate the expression of *NQO1*, *GCLC*, and *HMOX1*, which are involved in Nrf2-mediated antioxidant activity [37], neither propofol nor muscimol affected the expression of these genes under our experimental conditions. Nrf2 can suppress IL-6 and IL-1*β* production without activating the expression of *NQO1*, *GCLC*, or *HMOX1* during M1 polarization of human macrophages. Further investigation is needed to determine the precise molecular mechanisms by which Nrf2 selectively regulates the expression of genes encoding inflammatory molecules.

Surgical trauma can induce systemic acute-phase responses (APRs) and elevate levels of acute-phase proteins (APPs). Postsurgical inflammation is mediated by inflammatory cytokines, which are activated during early responses to tissue injury. IL-6 is primarily responsible for inducing APRs in the liver, including the production of C-reactive protein (CRP) and other APPs, and plays a major role in inflammation [7, 38–40]. Hepatic APP expression in response to LPS has been reported to be dependent on IL-6, but not on TNF-*α* [41]. IL-6 and CRP concentrations are regarded as useful clinical markers to reflect the extent of direct surgical tissue injury, postoperative inflammatory state, and degree of host defense mechanisms [42, 43]. Inhibition of IL-6 production may suppress systemic inflammation induced by surgical trauma, thereby reducing postoperative complications.

In conclusion, the present study showed that propofol suppresses IL-6 and IL-1*β* expression during human M1 macrophage polarization, suggesting that propofol plays a protective role in the development and progression of inflammation. The GABA_A_ receptor- and Nrf2-mediated signal transduction pathway is thought to be involved in the inhibitory effects of propofol. These findings can help clarify the molecular mechanisms by which propofol suppresses inflammatory responses.

## Figures and Tables

**Table 1:** Primer sequences for quantitative real-time RT-PCR.

Gene	Forward primer (5′ → 3′)	Reverse primer (5′ → 3′)
*β*-Actin	TGGCACCCAGCACAATGAA	CTAAGTCATAGTCCGCCTAGAAGCA
IL-6	AAGCCAGAGCTGTGCAGATGAGTA	TGTCCTGCAGCCACTGGTTC
IL-1*β*	CCAGGGACAGGATATGGAGCA	TTCAACACGCAGGACAGGTACAG
TNF-*α*	GACAAGCCTGTAGCCCATGTTGTA	CAGCCTTGGCCCTTGAAGA
IL-10	GAGATGCCTTCAGCAGAGTGAAGA	AGGCTTGGCAACCCAGGTAAC
TGF-*β*1	AGCGACTCGCCAGAGTGGTTA	GCAGTGTGTTATCCCTGCTGTCA
CD86	CTGTAACTCCAGCTCTGCTCCGTA	GCCCATAAGTGTGCTCTGAAGTGA
CD206	GCCCGGAGTCAGATCACACA	AGTGGCTCAACCCGATATGACAG
NQO1	GGATTGGACCGAGCTGGAA	GAAACACCCAGCCGTCAGCTA
HMOX1	TTGCCAGTGCCACCAAGTTC	TCAGCAGCTCCTGCAACTCC
GCLC	GTCCACAAATTGGCAGACAATGA	ACTCTGGTGAGCAGTACCACAAACA

## Data Availability

The data used to support the findings of this study are available from the corresponding author upon request.

## Supplementary Materials

Supplementary 1 Supplementary Figure 1: expression of M1 and M2 macrophage markers during polarization of M0 macrophages. (A) qRT-PCR assays of CD86, CD206, IL-6, IL-1*β*, and TNF-*α* mRNA levels. Data were normalized relative to *β*-actin mRNA (internal control) and presented as mean ± SD (*n* = 3 per group). (B) Flow cytometric analysis of surface expression of CD86 and CD206 on M0 (thin line) and M1 (thick line) macrophages and isotype-matched control IgGs for M0 (gray fill) and M1 (dashed line) macrophages. (C) Flow cytometric analysis of surface expression of CD86 and CD206 on M0 (thin line) and M2 (thick line) macrophages and isotype-matched control IgGs for M0 (gray fill) and M2 (dashed line) macrophages.

Supplementary 2 Supplementary Figure 2: expression of M1 and M2 macrophage markers during polarization from M0 to M1 and M2 THP-1 cells. qRT-PCR assays of CD86 (A), CD206 (B), IL-6 (C), IL-1*β* (D), and TNF-*α* (E) mRNA levels. Data were normalized relative to *β*-actin mRNA (internal control) and presented as mean ± SD (*n* = 3 per group).

Supplementary 3 Supplementary Figure 3: propofol reduced IL-6 and IL-1*β* gene expression and protein production in M1 THP-1 cells. M0 THP-1 cells were polarized to M1 THP-1 cells in the presence of 0.05% DMSO as a solvent control (control) or propofol (25–100 *μ*M). (A-C) qRT-PCR assays of IL-6, IL-1*β*, and TNF-*α* mRNA in M1 THP-1 cells. Data were normalized relative to *β*-actin mRNA (internal control) and presented as mean ± SD (*n* = 3 per group). (D-F) Concentrations, determined by ELISA, of IL-6 (D), IL-1*β* (E), and TNF-*α* (F) secreted by M1 THP-1 cells. Data are presented as mean ± SD (*n* = 3 per group). ^∗^ *P* < 0.05, ^∗∗^ *P* < 0.01 compared with control by one-way ANOVA and Bonferroni's post hoc test.

Supplementary 4 Supplementary Figure 4: propofol had no effect on IL-10, TGF-*β*1, or CD206 gene expression and protein production in M2 THP-1 cells. M0 THP-1 cells were polarized to M2 THP-1 cells in the presence of 0.05% DMSO (control) or propofol (25–100 *μ*M). (A-C) qRT-PCR assays of IL-10, TGF-*β*1, and CD206 mRNA. ELISA measurements of IL-10 (D) and TGF-*β*1 (E), secreted by M2 macrophages. Data are presented as mean ± SD (*n* = 3 per group). Data were normalized relative to *β*-actin mRNA (internal control) and presented as mean ± SD (*n* = 3 per group). Data were analyzed using one-way ANOVA and Bonferroni's post hoc test.

Supplementary 5 Supplementary Figure 5: propofol and muscimol had no effect on NQO1, GCLC, or HMOX1 mRNA expression in M1 macrophages. M0 macrophages were polarized to M1 macrophages during treatment with 0.05% DMSO (control), propofol (1-5 *μ*M), or muscimol (100 *μ*M). (A-F) qRT-PCR assays of NQO1, GCLC, and HMOX1 mRNA. Data were normalized relative to *β*-actin mRNA (internal control) and presented as mean ± SD (*n* = 4 per group). Comparisons were analyzed using one-way ANOVA and Bonferroni's post hoc test or Wilcoxon-Mann-Whitney test.
